# Finerenone in Cardiorenal Disease: A Narrative Review of Molecular Mechanisms, Clinical Evidence, and Emerging Therapeutic Roles

**DOI:** 10.1007/s10557-026-07890-7

**Published:** 2026-05-22

**Authors:** Giulio Geraci, Nicola Sinatra, Valentina Paternò, Vincenzo Calabrese, Giuliano Cassataro, Giuseppe Cuttone, Calogero Geraci, Roberta Esposito, Costantino Mancusi, Giacomo Pucci, Luca Zanoli, Giuseppe Mulè, Riccardo Polosa, Pietro Ferrara, Caterina Carollo

**Affiliations:** 1 Department of Medicine and Surgery, “Kore” University of Enna, Enna, Italy; 2 Internal Medicine Unit, Hospital Umberto I, ASP Enna, Enna, Italy; 3 Nephrology and Dialysis Unit, “Paolo Borsellino” Hospital, Marsala, Italy; 4 Department of Medicine and Pulmonology, Fondazione Istituto “G. Giglio”, Cefalù, Italy; 5 Anesthesia and Intensive Care, “Abele Ajello” Hospital, Mazara del Vallo, ASP, Trapani, Italy; 6 Cardiology Unit, Provincial Health Authority of Caltanissetta (ASP), Caltanissetta, Italy; 7 Department of Clinical Medicine and Surgery, Federico II University Hospital, Naples, Italy; 8 Department of Advanced Biomedical Science, Federico II University of Naples, Naples, Italy; 9 Department of Medicine and Surgery, University of Perugia, Perugia, Italy; 10 Unit of Internal and Translational Medicine, Santa Maria Terni Hospital, Terni, Italy; 11 Department of Clinical and Experimental Medicine, University of Catania, Catania, Italy; 12 Unit of Internal Medicine, Department of Health Promotion, Mother and Child Care, Internal Medicine and Medical Specialties, University of Palermo, Palermo, Italy; 13 Centre of Excellence for the Acceleration of HArm Reduction (CoEHAR), University of Catania, Catania, Italy; 14 Center for Public Health Research, University of Milan–Bicocca, Via Cadore 48 – U38 Building Monza I, Monza, 20900 Italy; 15 Laboratory of Public Health, IRCCS Istituto Auxologico Italiano, Milan, Italy

**Keywords:** Finerenone, Mineralocorticoid Receptor Antagonist, Cardiorenal Syndrome, Diabetic Kidney Disease, Heart Failure, Chronic Kidney Disease, SGLT2 Inhibitors

## Abstract

Cardiorenal disease reflects a tightly interconnected pathophysiological continuum driven by neurohormonal activation, inflammation, oxidative stress, and progressive fibrosis. Among these mechanisms, mineralocorticoid receptor (MR) overactivation has emerged as a central mediator of cardiac and renal injury independent of blood pressure effects. Finerenone, a non-steroidal selective MR antagonist, exhibits distinct pharmacological properties compared with steroidal MR antagonists, including enhanced receptor selectivity, balanced cardiac and renal tissue distribution, and differential cofactor modulation that translates into potent anti-inflammatory and antifibrotic activity with an improved safety profile.

Large outcome trials have established finerenone as an effective cardiorenal protective therapy. The FIDELIO-DKD and FIGARO-DKD trials demonstrated significant reductions in kidney disease progression and cardiovascular events in patients with type 2 diabetes and chronic kidney disease, with consistent benefits confirmed in the pooled FIDELITY analysis. More recently, the FINEARTS-HF trial extended these benefits to patients with heart failure with mildly reduced or preserved ejection fraction regardless of diabetes status. Across trials, hyperkalemia was infrequent when structured monitoring strategies were applied.

Beyond established indications, emerging data suggest potential systemic effects on retinal and hepatic outcomes, while ongoing studies are evaluating finerenone in non-diabetic chronic kidney disease. Subgroup analyses consistently demonstrate preserved efficacy and favorable safety when finerenone is combined with sodium–glucose cotransporter-2 inhibitors, supporting complementary disease-modifying strategies.

This review integrates molecular mechanisms, clinical trial evidence, systemic effects, and therapeutic positioning of finerenone within contemporary cardiorenal care, highlighting its expanding role in multidrug approaches targeting the cardiovascular–renal–metabolic axis.

## Introduction

### The Cardiorenal Continuum: Epidemiology and Pathophysiological Interplay

The intricate interplay between cardiovascular and renal diseases has emerged as one of the most compelling areas of clinical research in the past two decades, fundamentally reshaping contemporary concepts of organ crosstalk and therapeutic strategies. The formal classification of cardiorenal syndrome, firstly introduced by Ronco and colleagues in 2008, recognizes the bidirectional nature of heart-kidney interactions, whereby acute or chronic dysfunction in one organ directly promotes dysfunction in the other [1]. This conceptual shift has moved beyond the traditional view of cardiac and renal diseases as isolated entities, establishing them instead as interconnected components of a pathophysiological continuum that shares common risk factors, hemodynamic interdependence, and sustained neurohormonal activation [2]. The global burden of cardiorenal disease continues to rise, with chronic kidney disease (CKD) affecting approximately 10–15% of the adult population worldwide and representing both a consequence and a powerful predictor of cardiovascular morbidity and mortality [3]. Individuals with CKD experience a cardiovascular mortality risk 10 to 30 times higher than that of the general population, and most die from cardiovascular causes before reaching end-stage renal disease [4]. This striking epidemiological overlap has intensified the search for therapeutic strategies capable of simultaneously protecting cardiac and renal function, particularly in high-risk populations such as those with type 2 diabetes mellitus (T2DM), in whom metabolic, hemodynamic, and inflammatory pathways converge to accelerate organ damage [5].

The pathophysiology of cardiorenal disease reflects a complex network of interconnected mechanisms that extend well beyond traditional hemodynamic perturbations. At the core of this process lies chronic overactivation of the renin-angiotensin-aldosterone system (RAAS), which promotes sodium retention, potassium imbalance, endothelial dysfunction, oxidative stress, inflammation, and progressive fibrosis in both cardiac and renal tissues [6]. Within this cascade, mineralocorticoid receptor (MR) activation – primarily driven by aldosterone and cortisol – has emerged as a pivotal mediator of tissue injury. The recognition that MR overactivation drives cardiovascular and renal damage independently of blood pressure effects has opened new therapeutic avenues, positioning MR antagonism as an important component of contemporary cardiorenal protection, with the strongest clinical evidence currently established in patients with T2DM and CKD [7].

### Evolution of Cardiorenal Therapies and the Emergence of Finerenone as a Next-Generation Mineralocorticoid Receptor Antagonist

The therapeutic landscape for cardiorenal protection has evolved profoundly over the past decade, expanding from a primary reliance on of RAAS blockade with angiotensin-converting enzyme inhibitors (ACEi) and angiotensin receptor blockers (ARB) to include novel pharmacological classes with broad pleiotropic effects. The advent of sodium-glucose cotransporter-2 (SGLT2) inhibitors represented a paradigm shift in cardiorenal medicine, as pivotal trials such as CREDENCE, DAPA-CKD, and EMPA-KIDNEY demonstrated robust reductions in both kidney disease progression and cardiovascular events across diabetic and non-diabetic populations [8–10]. Collectively, these findings have refined standard of care and underscored the clinical value of targeting multiple converging pathophysiological pathways.

Within this evolving framework, finerenone has emerged as a particularly promising agent, representing a new generation of nonsteroidal, selective MR antagonists with pharmacological properties that clearly distinguish it from traditional steroidal MR antagonists such as spironolactone and eplerenone [11]. In contrast to earlier agents, finerenone exhibits greater specificity for MR, more balanced distribution across cardiac and renal tissues, and a distinct molecular mechanism characterized by differential cofactor recruitment and gene transcription modulation [12]. These features translate into enhanced antifibrotic and anti-inflammatory activity with an improved safety profile, particularly regarding a lower propensity for clinically significant hyperkalemia – a key limitation of steroidal MR antagonists in CKD patients [13]. The key pharmacological characteristics, pivotal clinical trials, and current therapeutic indications of finerenone are summarized in Table 1.

**Table 1:** Pharmacological and Clinical Characteristics of Mineralocorticoid Receptor Antagonists: Finerenone Versus Steroidal Mineralocorticoid Receptor Antagonists

Characteristic	Finerenone	Spironolactone	Eplerenone
Chemical structure	Non-steroidal (dihydronaphthyridine core)	Steroidal	Steroidal
MR selectivity	High	Low (also binds androgen, progesterone, glucocorticoid receptors)	Moderate
Half-life	2–3 h	12–24 h (active metabolites)	4–6 h
Active metabolites	No	Yes (canrenone)	No
Main metabolic pathway	CYP3A4 (88–89%)	CYP3A4	CYP3A4
Renal elimination of unchanged drug	< 1%	Minimal	~ 5%
Heart/kidney tissue distribution	Balanced	Predominantly renal	Predominantly renal
Molecular mechanism	Blockade of SRC-1 recruitment, promotion of corepressors	Classical competitive antagonism	Classical competitive antagonism
Antifibrotic effects	Marked (↓TGF-β, ↓collagen I/III)	Moderate	Moderate
Anti-inflammatory effects	Marked (↓NF-κB, ↓IL-6, ↓TNF-α)	Moderate	Moderate
Blood pressure reduction	Modest (~ 3 mmHg systolic)	Marked	Moderate
Hyperkalemia incidence	21.4% (K⁺ >5.5), discontinuation 1.7–2.3%	Higher	Intermediate
Gynecomastia	0.1%	9–10%	< 1%
Approved indications	DKD with T2DM, HFmrEF/HFpEF	Heart failure, hyperaldosteronism, resistant hypertension, cirrhosis	Post-MI heart failure, HFrEF
Relevant drug interactions	Strong CYP3A4 inhibitors (contraindicated), moderate CYP3A4 inhibitors (dose adjustment)	CYP3A4 inhibitors	CYP3A4 inhibitors
Dose adjustment for hepatic impairment	Not required (Child-Pugh A-B)	Contraindicated in severe cirrhosis	Caution
Dose adjustment for renal impairment	Can be used down to eGFR 25 mL/min/1.73 m²	Limitations with reduced eGFR	Limitations with reduced eGFR

*Abbreviations: CYP3A4* cytochrome P450 3A4, *DKD* diabetic kidney disease, *HFpEF* heart failure with preserved ejection fraction, *HFmrEF* heart failure with mildly reduced ejection fraction, *HFrEF* heart failure with reduced ejection fraction, *MI* myocardial infarction, *MR* mineralocorticoid receptor, *RCT* randomized controlled trial, *SRC-1* steroid receptor coactivator-1, *T2DM* type 2 diabetes mellitus

The clinical development program for finerenone has generated robust and consistent evidence supporting its cardiorenal benefits. The pivotal FIDELIO-DKD and FIGARO-DKD trials demonstrated significant reductions in both renal and cardiovascular outcomes in patients with T2DM and CKD [14, 15]. More recently, the FINEARTS-HF trial extended these findings to patients with heart failure with mildly reduced or preserved ejection fraction (HFmrEF/HFpEF), irrespective of diabetes status, thereby broadening the therapeutic scope of finerenone across the cardiorenal continuum [16]. Collectively, these trials establish finerenone as an important component of contemporary cardiorenal therapy, with the strongest current clinical evidence supporting its use in patients with T2DM and CKD, while its role in broader heart failure populations continues to evolve.

The mechanisms underlying these clinical benefits extend beyond conventional MR blockade and encompass coordinated anti-inflammatory and antifibrotic effects. Preclinical investigations have shown that finerenone reduces the expression of pro-inflammatory cytokines, mitigates oxidative stress, and attenuates fibrotic signaling pathways in both cardiac and renal tissues [12, 13, 17]. These pleiotropic actions are mediated through both genomic and non-genomic pathways, with finerenone inducing distinct transcriptional cofactors recruitment compared with steroidal MR antagonists [18].

The translation of this evidence into clinical practice has been reinforced by the incorporation of finerenone into major international guidelines. The 2023 European Society of Cardiology guidelines for cardiovascular disease in diabetic patients assign a Class I, Level A recommendation to finerenone for patients with T2DM and CKD to reduce the risk of cardiovascular events and kidney disease progression [19]. Similarly, the 2024 Kidney Disease: Improving Global Outcomes (KDIGO) Clinical Practice Guideline recommends consideration of finerenone in patients with T2DM, CKD, and persistent albuminuria despite maximally tolerated doses of RAAS inhibition, particularly in those at high cardiovascular risk [20].

The potential for synergistic use of combining finerenone alongside other foundational cardiorenal therapies represents an important emerging frontier in contemporary clinical practice. Subgroup and pooled analyses from pivotal trials have demonstrated that the cardiorenal benefits of finerenone are maintained in patients receiving SGLT2 inhibitors, supporting complementary and potentially additive mechanisms of action [21]. The CONFIDENCE trial (NCT05254002) has been designed to prospectively evaluate the combined use of finerenone and empagliflozin in patients with CKD and T2DM, aiming to define the incremental benefits of dual pathway modulation [22]. In parallel, the FIND-CKD trial investigates the efficacy of finerenone in patients with non-diabetic CKD, potentially expanding its therapeutic scope beyond diabetic populations [23, 24].

### Scope and Objectives of the Review

This review provides a comprehensive overview of finerenone in cardiorenal medicine, integrating mechanistic insights, clinical trial evidence, emerging extracardiac effects, and evolving therapeutic strategies. By synthesizing current research, it aims to inform clinicians and researchers on the optimal integration of finerenone within multidrug approaches for cardiorenal protection and to highlight future research directions in this rapidly evolving field. The present work also aims to provide an updated and integrative perspective across the broader cardiorenal–metabolic axis. In particular, it combines mechanistic and clinical evidence, extends the discussion to emerging indications such as heart failure and non-diabetic CKD, and incorporates evolving data on systemic effects and combination strategies. By bridging molecular, translational, and clinical domains, this review offers a clinically oriented synthesis that goes beyond indication-specific summaries.

This narrative review was based on a non-systematic literature search conducted in PubMed/MEDLINE up to December 2025, using specific keywords including: “finerenone”, “mineralocorticoid receptor antagonist”, “cardiorenal”, “chronic kidney disease”, “diabetic kidney disease”, “heart failure”, “SGLT2 inhibitors”, and “hyperkalemia”. Priority was given to randomized controlled trials, pooled analyses, relevant mechanistic studies, and recent review articles. Additional references were identified through manual screening of the bibliographies of selected studies. Given the narrative nature of this review, formal systematic review methods, including predefined inclusion criteria and structured evidence synthesis, were not applied.

## Molecular Mechanisms of Finerenone: From Mineralocorticoid Receptor Antagonism to Cellular Signaling Pathways

Finerenone represents a major advance in MR antagonism, characterized by a unique nonsteroidal dihydronaphthyridine structure that confers high receptor selectivity and distinct pharmacokinetic and pharmacodynamic properties compared with steroidal MR antagonists spironolactone and eplerenone. In addition to its reduced off-target binding to androgen, progesterone, and glucocorticoid receptors, finerenone exhibits a more balanced distribution across cardiac and renal tissues, in contrast to the predominantly renal accumulation observed with steroidal agents [11, 13, 25]. This pharmacokinetic profile translates into a more homogeneous target engagement at both cardiac and renal levels, thereby enhancing its activity particularly within the complex pathophysiological framework of the cardio-kidney-metabolic syndrome.

**Fig. 1:**
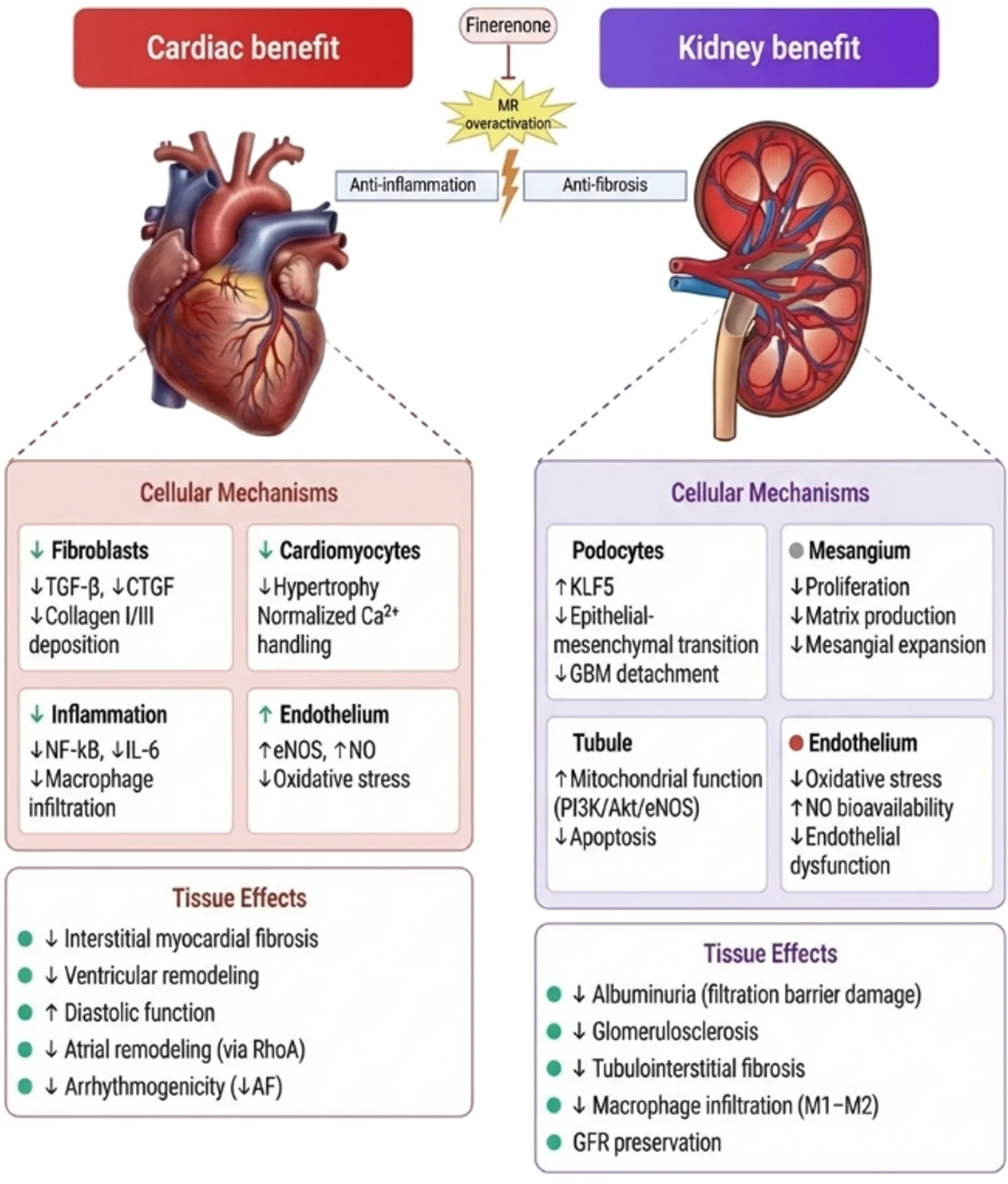
Molecular and cellular pathways underlying the cardiorenal protective effects of finerenone. Abbreviations: AF, atrial fibrillation, Ca2+, calcium; CTGF = connective tissue growth factor; eNOS, endothelial nitric oxide synthase; GBM, glomerular basement membrane; GFR, glomerular filtration rate; IL-6, interleukin-6; KLF5, Krüppel-like factor 5; MR, mineralocorticoid receptor; NF-κB, nuclear factor kappa B; NO, nitric oxide; PI3K, phosphoinositide 3-kinase; Akt, protein kinase B; RhoA, Ras homolog family member A; TGF-β, transforming growth factor beta; TNF-α, tumor necrosis factor alpha

The principal cellular and molecular mechanisms underlying finerenone-mediated cardiorenal protection are schematically illustrated in Fig. 1. At the molecular level, finerenone acts as a competitive MR antagonist but induces a distinct conformational change in the MR ligand-binding domain. Structural and biophysical studies have shown that this conformational profile impairs the recruitment of transcriptional coactivators – particularly steroid receptor coactivator-1 (SRC-1) – while promoting the engagement of corepressors complexes [18, 26]. This selective modulation of MR cofactor interaction results in more effective repression of pathogenic MR-driven gene transcription compared with steroidal antagonists, which behave as partial agonists under certain cellular contexts [26].

At the cellular level, finerenone modulates both genomic and non-genomic MR signaling pathways. Its genomic effects include inhibition of MR nuclear translocation and reduced binding to hormone response elements within target gene promoters, leading to suppression of transcriptional programs involved in inflammation, fibrosis, and oxidative stress [24]. Consistent with these effects, preclinical models have demonstrated robust antifibrotic activity of finerenone in both cardiac and renal tissues. In the kidney, finerenone limits the progression from acute kidney injury to CKD through attenuation of oxidative stress and downregulation of pro-fibrotic signaling cascades [27]. In cardiac tissue, antifibrotic activity is mediated in part through selective MR cofactor recruitment [26].

Anti-inflammatory actions of finerenone are mediated through multiple converging mechanisms. Experimental models of diabetic kidney disease (DKD) have demonstrated that finerenone suppresses nuclear factor-κB (NF-κB) activation, leading to reduced expression of pro-inflammatory cytokines including interleukin-6 (IL-6), tumor necrosis factor-α (TNF-α), and monocyte chemoattractant protein-1 (MCP-1) [28, 29]. Additionally, finerenone decreases macrophage infiltration and promotes a phenotypic shift from pro-inflammatory M1 macrophages toward anti-inflammatory M2 polarization in both experimental renal and cardiac injury models [18, 24]. The antifibrotic properties of finerenone extend to direct modulation of profibrotic signaling networks. Reduced transforming growth factor-β (TGF-β) expression and decreased collagen deposition have been observed in cardiac tissue, accompanied by transcriptomic changes in key inflammatory and fibrotic pathways [30]. In the kidney, finerenone interferes with epithelial-to-mesenchymal transition in podocytes via upregulation of Krüppel-like factor 5 (KLF5), a critical regulator of fibrogenesis [31].

Attenuation of oxidative stress represents a further central mechanism of finerenone-mediated organ protection. Finerenone enhances endogenous antioxidant defenses, including superoxide dismutase and catalase, while suppressing NADPH oxidase-dependent reactive oxygen species production [27, 32]. In endothelial cells, finerenone preserves nitric oxide bioavailability by preventing oxidative inactivation and maintaining endothelial nitric oxide synthase expression, thereby improving vascular function and reducing albuminuria [28, 33].

Beyond structural remodeling, MR signaling is increasingly recognized as a contributor to metabolic dysfunction. Preclinical studies have demonstrated that MR blockade improves insulin sensitivity through enhanced adipose tissue insulin signaling and suppression of hepatic gluconeogenesis, findings corroborated by clinical evidence demonstrating improved insulin resistance with MR antagonist therapy in individuals with obesity [34].

Recent evidence suggests that finerenone preserves cellular energetics through modulation of autophagy and mitochondrial homeostasis. In renal tubular epithelial cells, finerenone maintains mitochondrial dynamics by limiting excessive fission and promoting mitophagy of damaged mitochondria via activation the PI3K/Akt/eNOS pathway, thereby reducing apoptosis and preserving renal function [35]. Similarly, in cardiomyocytes, finerenone improves calcium handling through normalization of phospholamban and calcium/calmodulin-dependent protein kinase II phosphorylation, mitigating systolic and diastolic dysfunction in CKD [36]. These interconnected molecular actions position finerenone not merely as a mineralocorticoid receptor blocker but as a broad modulator of inflammatory, fibrotic, oxidative, metabolic, and mitochondrial pathways central to cardiorenal disease progression.

## Finerenone in Diabetic Kidney Disease: Evidence from Landmark Trials and Guidelines Integration

The clinical development program of finerenone in DKD represents one of the most comprehensive therapeutic evaluations in contemporary nephrology, reshaping the management paradigm for this high-risk population. The phase 2b ARTS-DN trial first demonstrated proof of concept, enrolling 823 patients with T2DM and CKD and showing dose-dependent reductions in albuminuria of up to 38% at 90 days with an acceptable safety profile, thereby establishing the rationale for large-scale outcome trials [37].

The pivotal FIDELIO-DKD trial subsequently enrolled 5,734 patients with T2DM and CKD at high renal risk. Eligibility requires an eGFR of 25 to < 60 mL/min/1.73m^2^ with moderately increased albuminuria (UACR 30–<300 mg/g) in the presence of diabetic retinopathy, or an eGFR of 25 to < 75 mL/min/1.73 m² with severely increased albuminuria (UACR 300–5,000 mg/g) [14]. Over a median follow-up of 2.6 years, finerenone reduced the primary composite kidney outcome – kidney failure, sustained ≥ 40% decline in eGFR, or renal death – by 18% (hazard ratio [HR] 0.82, 95% CI 0.73–0.93). The key secondary cardiovascular composite endpoint – cardiovascular death, nonfatal myocardial infarction, nonfatal stroke, or HF hospitalization – was reduced by 14% (HR 0.86, 95% CI 0.75–0.99). Importantly, treatment effects were consistent across prespecified subgroups, including baseline eGFR strata, albuminuria categories, and background SGLT2 inhibitor use [14].

Complementing these findings, the FIGARO-DKD trial enrolled 7,437 patients with T2DM and a broader range of kidney function, including earlier–stage CKD, with a primary focus on cardiovascular outcomes [15]. Finerenone reduced the primary composite cardiovascular endpoint by 13% (HR 0.87, 95% CI 0.76–0.98), driven predominantly by a 29% reduction in first hospitalization for HF (HR 0.71, 95% CI 0.56–0.90). Although the kidney composite defined by ≥ 40% eGFR decline did not reach statistical significance (HR 0.87, 95% CI 0.76–1.01), a more stringent endpoint – ≥57% eGFR decline, kidney failure, or renal death – was significantly reduced by 23% (HR 0.77, 95% CI 0.60–0.99), including a 36% relative reduction in end-stage kidney disease [38].

The prespecified FIDELITY pooled analysis of FIDELIO-DKD and FIGARO-DKD, encompassing 13,026 patients with a median follow-up of 3.0 years, provided robust statistical power to evaluate finerenone’s effects across the cardiorenal continuum [39]. Finerenone reduced the composite cardiovascular outcome by 14% (HR 0.86, 95% CI 0.78–0.95) and the composite kidney outcome by 23% (HR 0.77, 95% CI 0.67–0.88). In on-treatment analyses, finerenone was associated with a significant reduction in all-cause mortality by 18% (HR 0.82, 95% CI 0.70–0.96) and cardiovascular mortality by 18% (HR 0.82, 95% CI 0.67–0.99), with a 25% reduction in sudden cardiac death (HR 0.75, 95% CI 0.57–0.996) [40].

Prespecified and post hoc subgroup analyses confirmed consistent efficacy across diverse clinically relevant populations. The cardiorenal benefits of finerenone were maintained in patients receiving baseline SGLT2 inhibitor, with no evidence of treatment interaction for cardiovascular or kidney outcomes (P interaction = 0.46 and = 0.29, respectively) [21]. Among individuals with more advanced renal impairment (baseline estimated glomerular filtration rate [eGFR] < 45 mL/min/1.73m^2^), finerenone reduced the kidney composite by 30% and slowed annual eGFR decline by 1.11 mL/min/1.73m^2^ [41]. Notably, cardiovascular benefits persisted in patients with normoalbuminuria (urine albumin-to-creatinine ratio [UACR] < 30 mg/g), representing 38% of FIGARO-DKD participants, challenging traditional albuminuria-based treatment paradigms.

Safety analyses demonstrated finerenone’s favorable tolerability profile. Hyperkalemia leading to treatment discontinuation occurred in only 2.3% of finerenone-treated patients versus 0.9% with placebo, with no hyperkalemia-related deaths [42]. Rates of acute kidney injury were numerically lower with finerenone (6.6% vs. 7.5%), and endocrine adverse effects such gynecomastia rates were rare (0.1%), contrasting markedly with steroidal MR antagonists [39, 43]. Mode-of-death analyses further revealed significant reductions in sudden cardiac death (–21%) HF-related death (–27%), supporting potential antiarrhythmic and anti-remodeling effects [44].

Based on this compelling evidence, finerenone has been rapidly incorporated into international clinical practice guidelines. The 2022 American Diabetes Association Standards of Care recommend finerenone for patients with T2DM and CKD at elevated risk for cardiovascular or renal risk (Level A evidence), as well as for those who are unable to use SGLT2 inhibitors [45]. The 2024 KDIGO guideline endorses nonsteroidal MR antagonists for patients with T2DM, eGFR ≥ 25 mL/min/1.73m^2^, normal serum potassium, and persistent despite optimized RAAS blockade [20]. Similarly, the 2023 European Society of Cardiology guidelines assign a Class I, Level A recommendation for finerenone in eligible patients with DKD. Finerenone is recommended in addition to an ACEi or ARB in patients with T2DM and eGFR > 60 mL/min/1.73 m^2^ with a UACR ≥ 30 mg/mmol (≥ 300 mg/g), or eGFR 25–60 mL/min/1.73 m^2^ and UACR ≥ 3 mg/mmol (≥ 30 mg/g) to reduce cardiovascular events and kidney failure [19].

**Fig. 2:**
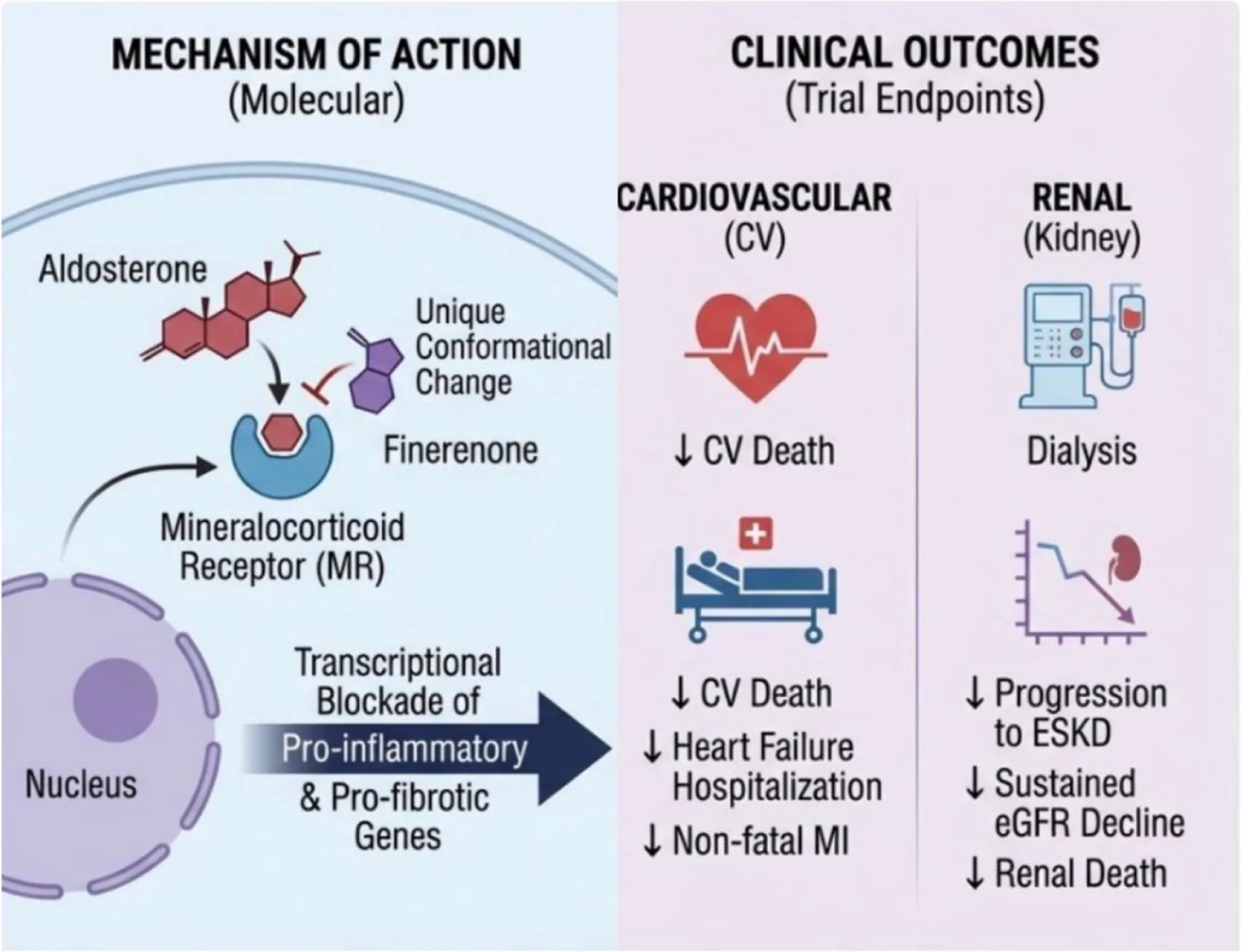
Translational bridge of finerenone: from molecular blockade to cardiorenal clinical benefits

Taken together, these findings support the role of finerenone in DKD, which currently represents the clinical setting with the most robust and consistent evidence base. In this context, finerenone is associated with clinically meaningful cardiorenal protection aligned with the pathophysiological continuum of diabetic complications. The clinical evidence supporting finerenone across the cardiorenal continuum is summarized in Fig. 2.

## Cardiovascular Effects of Finerenone: Impact on Heart Failure, Fibrosis, and Vascular Function

The cardiovascular benefits of finerenone extend beyond DKD to encompass a broader HF population, as demonstrated by its comprehensive clinical trial program. The FINEARTS-HF trial provides definitive evidence for finerenone in patients with HFmrEF/HFpEF, enrolling 6,001 symptomatic individuals with left ventricular ejection fraction of at least 40% [16]. Over a median follow-up of 32 months, finerenone reduced the primary composite outcome of cardiovascular death and total worsening HF events by 16% (rate ratio [RR] 0.84, 95% CI 0.74–0.95). This effect was driven predominantly by a reduction in worsening HF events (RR 0.82, 95% CI 0.71–0.94), while cardiovascular mortality showed a non-significant downward trend (HR 0.93, 95% CI 0.78–1.11). Importantly, treatment benefits were consistent across the full spectrum of ejection fractions and irrespective of baseline diabetes status, supporting finerenone as a promising therapeutic option for HFmrEF/HFpEF, although its clinical role in heart failure remains less established than in DKD and is primarily informed by more recent trial evidence. The safety profile in FINEARTS-HF was favorable, with lower rates of hyperkalemia-related discontinuation occurring compared with historical experience with steroidal MR antagonists [13]. In addition, finerenone was associated with significant improvements in patient-reported quality of life improvements as assessed by the Kansas City Cardiomyopathy Questionnaire.

The mechanistic basis for finerenone’s cardiovascular protection is closely linked to its antifibrotic within the myocardium. In preclinical models, MR antagonism attenuates markers of myocardial fibrosis and limits extracellular matrix remodeling, and ongoing imaging sub-analyses aim to confirm these structural effects in clinical settings [29]. In HF, MR antagonists have consistently been associated with favorable cardiac remodeling, including improvements in diastolic function and left atrial size [46]. These mechanisms are particularly relevant in HFpEF, characterized by myocardial stiffness and interstitial fibrosis.

Complementary clinical evidence derives from FIGARO-DKD, in which finerenone reduces new-onset HF by 32% (HR 0.68, 95% CI 0.50–0.93) and first HF hospitalizations by 29% (HR 0.71, 95% CI 0.56–0.90) [47] in patients with diabetic CKD. Importantly, these benefits were consistent irrespective of baseline HF history, supporting a preventive as well as therapeutic role across the spectrum of disease severity.

Finerenone also appears to confer antiarrhythmic benefits. A post-hoc analysis of FIDELIO-DKD demonstrated a 29% reduction in new-onset atrial fibrillation (HR 0.71, 95% CI 0.53–0.94) [44]. Mechanistically, MR activation promotes atrial fibrotic remodeling through Ras homolog family member A (RhoA) signaling, connective tissue growth factor (CTGF) upregulation, and enhanced lysyl oxidase expression – pathways attenuated by MR antagonists [48]. Reduction of myocardial fibrosis may therefore contribute not only to improved hemodynamics but also to electrical stability.

Earlier supportive evidence was provided by the ARTS-HF trial, which enrolled 1,066 patients with worsening heart failure with reduced ejection fraction and concomitant diabetes and/or CKD [49]. Finerenone demonstrated similar efficacy to eplerenone in reducing N-terminal pro–B-type natriuretic peptide (NT-proBNP) levels, with the 10→20 mg dose achieving a significant reduction in the composite clinical endpoint (HR 0.56, 95% CI 0.35–0.90) [50].

Additional preclinical studies further support a finerenone’s multifaceted cardiovascular mechanism. In murine models of CKD, MR antagonism improved diastolic dysfunction through normalization of calcium handling proteins [36], while selective modulation of MR cofactor recruitment resulted in differential transcriptional regulation compared with steroidal MR antagonists [26].

The convergence of antifibrotic, anti-remodeling, and antiarrhythmic effects – coupled with consistent outcome reductions across FIDELIO-DKD, FIGARO-DKD, and FINEARTS-HF – supports a role for finerenone within the broader cardiorenal therapeutic approach, with consistent evidence in DKD and emerging data in heart failure populations. Its cardiovascular benefits, maintained even in the presence of background SGLT2 inhibitor therapy, support integration into contemporary multidrug, guideline-directed strategies for patients across the cardiorenal spectrum.

## Renal Protection Beyond Diabetes: Finerenone in Non-Diabetic Chronic Kidney Disease

Although the efficacy of finerenone in DKD is well-established through FIDELIO-DKD and FIGARO-DKD, clinical evidence in non-diabetic CKD remains limited. The pathophysiological rationale for MR antagonism extends beyond diabetic nephropathy, as aldosterone-driven signaling promotes renal inflammation, fibrosis, and podocyte injury across different etiologies [51]. Consistent with this concept, meta-analyses of steroidal MR antagonists (i.e., spironolactone, eplerenone) have demonstrated antiproteinuric effects in heterogeneous CKD populations including non-diabetic etiologies [52]. Dedicated trials of finerenone in non-diabetic CKD, notably FIND-CKD and FINEGEN in Alport syndrome (NCT05047263), are currently underway and are expected to clarify its therapeutic role in these settings [23, 24].

In IgA nephropathy – the most common primary glomerulonephritis worldwide – MR antagonism represents a plausible therapeutic strategy, although no finerenone-specific trials have not yet been reported. Preclinical evidence suggests that MR activation contributes to mesangial injury and fibrosis in glomerulonephritis models, providing mechanistic support for future clinical investigation [53].

In autosomal dominant polycystic kidney disease (ADPKD), tolvaptan remains the only disease-modifying therapy with proven to slow kidney volume expansion and eGFR decline [54, 55]. The potential role of MR signaling to cyst progression and interstitial fibrosis has not been systematically explored, and no clinical trials of finerenone in ADPKD have been registered to date.

Similarly, therapeutic options for focal segmental glomerulosclerosis (FSGS) – a podocytopathy characterized by severe proteinuria and variable progression – remain limited. Preclinical studies implicate MR activation in podocyte injury and proteinuria through Rac1-mediated pathways [56], providing theoretical rationale for MR antagonist use. However, clinical evaluation of finerenone in FSGS has not yet been undertaken, and ongoing development of targeted agents such as sparsentan may reshape the treatment landscape [57].

In lupus nephritis, recent advances have focused on B-cell targeting (i.e., belimumab, obinutuzumab) and calcineurin inhibition (i.e., voclosporin), while the potential role of MR antagonism is largely unexplored. At present, no clinical trials of finerenone in this setting are registered or ongoing.

In resistant hypertension, MR antagonism has proven highly effective, with the PATHWAY-2 trial demonstrating spironolactone as the most effective fourth-line agent for blood pressure reduction compared with bisoprolol and doxazosin as [58]. In contrast, finerenone has not been specifically studied in resistant hypertension or hypertensive nephropathy. Subgroup analyses from FIDELIO-DKD and FIGARO-DKD suggest only modest antihypertensive effects (approximately 3 mmHg systolic reduction), supporting the concept that the primary benefits of finerenone appear to be independent of blood pressure lowering. Beyond outcome trials in diabetic kidney disease, mineralocorticoid receptor antagonism has long been recognized as a key therapeutic strategy in resistant hypertension and hypertensive organ damage, further supporting the pathophysiological relevance of MR signaling in cardiorenal disease [59].

Real-world evidence for finerenone remains limited. While post-marketing data in DKD populations are beginning to accumulate, no published observational studies have evaluated its use in non-diabetic CKD. In the absence of randomized outcome data, real-world studies will be essential to characterize effectiveness, safety, and patterns of off-label use in broader CKD populations.

Current safety data for finerenone derive exclusively from DKD and HF populations. In clinical trials, hyperkalemia emerged as the primary adverse event, although structured potassium monitoring and dose-adjustment algorithms effectively minimized treatment discontinuation. The generalizability of these safety findings to non-diabetic CKD remains unknown. Guidance from the KDIGO Controversies Conference on potassium homeostasis emphasizes individualized monitoring strategies for patients receiving RAAS inhibitors and MR antagonists [60]. Until population-specific data become available, clinicians considering off-label finerenone use in non-diabetic CKD should adhere to the rigorous monitoring protocols employed in the pivotal trials.

## Systemic Effects of Finerenone

Mineralocorticoid receptors are widely expressed in multiple tissues beyond the heart and kidney, including the retinal pigment epithelium, hepatic stellate cells, lung epithelium, and adipose tissue. Experimental evidence indicates that MR activation promotes to inflammation and fibrosis in these organs, supporting the hypothesis that MR antagonists might confer multiorgan protection. Beyond its established cardiorenal benefits, finerenone has therefore been explored for potential systemic effects extending to extracardiac and extrarenal tissues.

Current evidence suggests that finerenone may exert beneficial effects beyond the heart and kidney. Exploratory analyses point to a potential protective role in diabetic retinopathy, although confirmation in adequately powered randomized trials is required. Finerenone also appears safe in patients with hepatic steatosis and fibrosis, with neutral effects on liver enzymes and preserved cardiorenal efficacy; notably, cardiovascular benefits may be more pronounced in individuals with higher fibrosis-4 (FIB-4) scores. However, available data are derived primarily from subgroup and post hoc analyses rather than dedicated randomized studies. Prospective trials with sufficient statistical power are therefore needed to establish the clinical relevance of finerenone’s systemic effects.

### Retina

Activation of the MR-aldosterone axis contributes to retinal vascular dysfunction and inflammatory remodeling relevant to the pathogenesis of diabetic retinopathy. In preclinical models of diabetic and neovascular retinopathy, finerenone reduced microvascular injury and increased regulatory T-cell infiltration, suggesting a protective effect on retinal microvasculature [61]. However, these findings are biologically plausible, the available evidence is derived from exploratory and secondary analyses and should be interpreted cautiously. The retina and kidney share embryological, structural, and microvascular features, suggesting that systemic microvascular dysregulation can manifest in both organs. Indeed, retinal vascular changes have been associated with early renal dysfunction in hypertensive patients, indicating a broader microvascular link between the eye and kidney that may be relevant to cardiorenal pathology and potentially to therapies targeting shared mechanisms such as RAAS activation [62, 63].

Clinical insights derive from ReFineDR/DeFineDR, a pooled retrospective analysis of ophthalmological data from patients with non-proliferative diabetic retinopathy (NPDR) enrolled in the FIDELIO-DKD and FIGARO-DKD trials [64]. Among 244 patients (134 finerenone, 110 placebo), the occurrence of vision-threatening complications (VTCs), defined as anterior segment neovascularization, diabetic macular oedema, or progression to proliferative diabetic retinopathy, was numerically lower in the finerenone group at 2 years (3.7% vs. 6.4%; difference − 2.6%, 95% CI − 8.2% to 2.9%). At 36 months, the difference reached statistical significance (− 11.8%, 95% CI − 22.9% to − 0.7%), with consistent trends across subgroups stratified by baseline glicemic control, NPDR severity, and diabetes duration.

Notwithstanding these encouraging findings, important methodological limitations warrant caution. The retrospective design, limited sample size, and baseline imbalances favoring the finerenone group, and the reliance on non-standardized routine ophthalmological examinations preclude definitive casual inference. Accordingly, the authors appropriately characterize these results as hypothesis-generating, underscoring the need for prospective randomized trials to confirm whether finerenone can delay NPDR progression. If confirmed, finerenone could represent a novel oral therapeutic option in a disease currently managed predominantly with invasive interventions at advanced stages.

### Liver

The efficacy and safety of finerenone in patients with markers of hepatic steatosis and fibrosis have been evaluated in a prespecified subgroup analysis of the FIDELITY population [65]. This post-hoc evaluation stratified 13,026 patients according to hepatic steatosis index (> 36), alanine transaminase levels (> 33 IU/L in males, > 25 IU/L in females), and FIB-4 index scores (> 1.30, > 2.67, > 3.25). Across the strata, liver enzymes – including ALT, AST, and gamma-glutamyl transferase – remained stable over time and were comparable between finerenone and placebo groups, indicating a neutral hepatic safety profile. Importantly, finerenone was consistently associated with reductions in the composite kidney outcome irrespective of baseline liver test abnormalities. Cardiovascular risk increased progressively with higher FIB-4 scores; however, finerenone significantly lowered the composite cardiovascular outcome versus placebo across fibrosis strata: by 52% in patients with FIB-4 > 3.25, by 39% in those with FIB-4 > 2.67, and by 24% in those with FIB-4 > 1.30 (p for interaction = 0.01, 0.13, and 0.03, respectively). It should be noted that these findings are derived from post hoc subgroup analyses and are not based on trials specifically designed to assess hepatic outcomes, and should therefore be interpreted as hypothesis-generating pending confirmation in dedicated prospective studies. Pharmacokinetic investigations further support the hepatic safety of finerenone [66]. In moderate hepatic impairment (Child-Pugh class B), total and unbound area under the curve (AUC) increased by 38% and 55%, respectively, while peak plasma concentration (Cmax) remained unchanged. No clinically meaningful exposure changes were observed in mild hepatic impairment (Child-Pugh class A). Given the modest magnitude of these alterations and the lack of effects on peak concentrations, dose adjustment is not considered necessary in mild to moderate hepatic dysfunction.

To date, no randomized trials have investigated finerenone as a target treatment for hepatic steatosis or steatohepatitis. The current data support the safety of finerenone in metabolic dysfunction-associated steatotic liver disease and suggest preserved cardiorenal efficacy, particularly among patients with higher fibrosis risk, while direct hepatoprotective effects remain to be established.

### Other Organ Systems

At present, clinical evidence supporting finerenone effects on pulmonary, metabolic, or additional organ systems is lacking. Existing trials were not designed to assess these outcomes, and no dedicated substudies or registered clinical trials targeting extracardiac indications such as pulmonary fibrosis are available. Although mechanistic data suggest potential anti-inflammatory and antifibrotic actions across tissues, their clinical relevance beyond the heart and kidney remains speculative and warrants future investigation.

## Safety Profile and Clinical Considerations: Hyperkalemia Management and Drug Interactions

The clinical use of finerenone requires careful consideration of its safety profile, with hyperkalemia representing the primary safety concern requiring structured monitoring and management strategies. The FIDELIO-DKD and FIGARO-DKD trials established comprehensive potassium surveillance protocols that have become the standard for clinical practice, demonstrating that, with appropriate monitoring and dose-adjustment protocols, clinically significant hyperkalemia can be effectively managed [14]. In the pooled FIDELITY analysis, permanent discontinuation due hyperkalemia occurred in 1.7% of finerenone-treated patients compared with 0.6% in the placebo group [39]. However, hyperkalemia remains a well-recognized class effect of MR antagonism and requires structured potassium surveillance [14], particularly in patients with impaired kidney function or elevated baseline potassium levels.

The pharmacokinetic profile of finerenone contributes to its comparatively favorable hyperkalemia risk relative to steroidal MR antagonists. The drug exhibits a short half-life of approximately 2–3 h and lacks active metabolites, allowing for more rapid reversal of potassium elevations following dose adjustment or discontinuation than spironolactone, whose active metabolites persist for 12–24 h, or eplerenone, with half-life 4–6 of hours [11]. In patients with renal impairment, pharmacokinetic studies have demonstrated increases of approximately 50–60% in unbound area under the concentration–time curve, while peak plasma concentrations remain largely unchanged [67]. Together with minimal renal excretion of unchanged drug, these characteristics support predictable exposure and facilitate standardized potassium monitoring in clinical practice.

Risk factors for hyperkalemia during finerenone therapy have been characterized through a post hoc safety analysis of the FIDELIO-DKD trial [42]. Over a median follow-up of 2.6 years, treatment-emergent hyperkalemia (serum potassium > 5.5 mmol/L) occurred in 21.4% of finerenone-treated patients compared with 9.2% in the placebo group, while moderate hyperkalemia (> 6.0 mmol/L) occurred in 4.5% versus 1.4%, respectively. Multivariate Cox regression identified higher baseline serum potassium, lower eGFR, increased UACR, younger age, female sex, β-blocker use, and finerenone exposure as independent predictors of hyperkalemia. Conversely, concomitant diuretic and SGLT2 inhibitor use was associated with a lower observed risk of hyperkalemia in subgroup analyses [42]; however, these findings should be interpreted cautiously, as they are derived from post hoc analyses and have not been consistently replicated across all datasets. Early post-initiation increases in serum potassium and declines in eGFR predicted subsequent hyperkalemia in both treatment groups; however, for comparable short-term changes, the associated risk increase was smaller in the finerenone group than in placebo at month 4, suggesting adaptive potassium handling over time. The protective influence of concomitant SGLT2 inhibitor use has been further supported by pooled FIDELITY analyses [21]. Among 13,026 patients, 6.7% received an SGLT2 inhibitors at baseline and 8.5% initiated during follow-up. The cardiovascular and kidney benefits of finerenone were preserved irrespective of SGLT2 inhibitor use, with no significant treatment interaction for either outcome.

Comparative safety data further highlight the favorable hyperkalemia profile of finerenone relative to steroidal mineralocorticoid receptor antagonists. In the ARTS-HF trial, which compared finerenone with eplerenone in 1,066 patients with worsening heart failure and concomitant CKD and/or diabetes, potassium elevations ≥ 5.6 mmol/L occurred in only 4.3% of patients, with balanced incidence across finerenone dose groups and eplerenone [49]. Structured potassium monitoring protocols – incorporating dose adjustment and temporary treatment interruption for potassium levels exceeding 5.5 mmol/L – effectively mitigated clinical consequences and provide a pragmatic framework for routine use.

Beyond potassium-related effects, clinically relevant drug–drug interactions must be considered. Finerenone is primarily metabolized by CYP3A4, with minor involvement of CYP2C8, and exhibits an absolute bioavailability of approximately 43.5% due to first-pass metabolism. Co-administration with moderate CYP3A4 inhibitors substantially increased finerenone exposure, with erythromycin and verapamil raising area under the curve by 3.5-fold and 2.7-fold, respectively [68]. In contrast, inhibition of CYP2C8 with gemfibrozil had minimal impact on drug exposure, confirming that CYP3A4 accounts for nearly 90% of finerenone clearance. Accordingly, concomitant use with strong CYP3A4 inhibitors is contraindicated, while moderate inhibitors necessitate dose adjustment. Finerenone undergoes extensive hepatic metabolism with negligible renal excretion of unchanged drug (< 1%), and its metabolites lack MR activity [69].

The interaction profile of finerenone with other cardiorenal-protective agents has been systematically evaluated clinical trials and meta-analyses. In FINEARTS-HF, the clinical benefits of finerenone were preserved irrespective of concomitant SGLT2 inhibitor use (RR 0.83 [95% CI 0.60–1.16] with SGLT2 inhibitor vs. 0.85 [95% CI 0.74–0.98] without SGLT2 inhibitor; P for interaction = 0.76) [70]. Furthermore, a recent systematic review and meta-analysis incorporating both randomized and observational data confirmed that combined finerenone and SGLT2 inhibitor therapy significantly reduced all-cause mortality and major adverse kidney events compared with either agent alone, supporting additive cardiorenal protection through dual pathway modulation [71].

Evidence for combination with GLP-1 receptor agonists remains more limited but mechanistically compelling. Meta-regression analyses across cardiovascular outcome trials have demonstrated that the benefits of GLP-1 receptor agonists on major adverse cardiovascular events, cardiovascular mortality, and kidney outcomes are consistent irrespective of baseline SGLT2 inhibitor use (P for interaction > 0.05 for all endpoints) [72]. Although no dedicated trials have evaluated triple therapy with finerenone, SGLT2 inhibitors, and GLP-1 receptor agonists, convergent mechanistic rationale support further investigation of multi-agent approaches targeting complementary pathophysiological pathways.

Certain patient populations require individualized consideration, although dedicated subgroup analyses remain sparse. Pharmacokinetic studies have characterized finerenone exposure in hepatic impairment, informing dose recommendations according to Child–Pugh classification [73].

Strategies for managing hyperkalemia during finerenone therapy have been informed by the FIDELIO-DKD safety analysis, which identified modifiable risk factors and demonstrated that concomitant SGLT2 inhibitor or diuretic use reduced hyperkalemia risk [42]. In addition, novel potassium binders, including sodium zirconium cyclosilicate and patiromer, have shown efficacy in enabling continuation of RAAS inhibitors in hyperkalemic patients [74, 75], although specific data in finerenone-treated populations remain limited.

Finally, long-term safety data beyond trial follow-up periods and effectiveness in routine clinical practice are still being defined. Ongoing post-marketing surveillance and prospective real-world registries will be essential to validate the favorable safety profile observed in randomized trials across broader and more heterogeneous patient populations.

## Finerenone Within Multidrug Cardiorenal Therapy: Evidence and Clinical Positioning

Contemporary management of cardiorenal disease is increasingly based on a multidrug strategy targeting complementary pathophysiological pathways, including RAAS inhibition, SGLT2 inhibitors, and, more recently, GLP-1 receptor agonists.

Direct head-to-head randomized trials comparing finerenone with steroidal MR antagonists or SGLT2 inhibitors are lacking, representing a key gap in the evidence base and limiting the ability to define its relative therapeutic positioning. Network meta-analyses have attempted indirect comparisons, though their interpretability is limited by heterogeneity in trial populations, comparators, and outcome definitions [76].

With regards to the interplay between finerenone and SGLT2 inhibitors, in the pooled FIDELITY dataset, finerenone consistently reduced the composite cardiovascular outcome irrespective of baseline SGLT2 inhibitor use (HR 0.85 [95% CI 0.71–1.01] with SGLT2 inhibitor vs. 0.87 [95% CI 0.76–0.98] without; P for interaction = 0.46), with comparable findings for the kidney composite outcome (P for interaction = 0.29) [21]. Similarly, in FINEARTS-HF, finerenone’s clinical benefits were preserved regardless of concomitant SGLT2 inhibitor therapy (P for interaction = 0.76) [70].

From a mechanistic perspective, finerenone and SGLT2 inhibitors target partially distinct but complementary pathways within the cardiorenal–metabolic axis. Finerenone primarily attenuates MR-mediated inflammation, oxidative stress, and fibrosis, thereby limiting structural remodeling in both cardiac and renal tissues. In contrast, SGLT2 inhibitors exert predominantly hemodynamic, tubular, and metabolic effects, including reduction of intraglomerular pressure through restoration of tubuloglomerular feedback, improvement in myocardial energetics, and modulation of volume status.

This mechanistic complementarity provides a biological rationale for combination therapy, supporting the potential for additive cardiorenal protection. However, while subgroup and pooled analyses suggest preserved efficacy with dual therapy, the incremental benefit of combined treatment remains to be confirmed in dedicated prospective trials specifically designed to assess combination strategies. The ongoing CONFIDENCE trial (NCT05254002) is directly evaluating combination therapy with finerenone and empagliflozin versus either agent alone in patients with CKD and T2DM and is expected to inform optimal therapeutic sequencing and integration [22].

Safety data further suggest potential advantages of combination strategies. Some subgroup and post hoc analyses have suggested that concomitant SGLT2 inhibitor use may be associated with a lower risk of hyperkalemia during finerenone therapy [42], possibly related to the kaliuretic effects of this class; however, this observation has not been consistently demonstrated across studies and should be interpreted cautiously until confirmed in prospective trials specifically designed to assess this interaction.

Combination therapy with finerenone, SGLT2 inhibitors, and GLP-1 receptor agonists represents an emerging multidrug strategy targeting complementary pathophysiological pathways in cardiorenal disease. While RAAS inhibitors remain the therapeutic backbone, the addition of SGLT2 inhibitors provides hemodynamic and metabolic benefits, and GLP-1 receptor agonists contribute to glycemic control and cardiovascular risk reduction. In this context, finerenone further attenuates inflammation and fibrosis mediated by mineralocorticoid receptor overactivation. Evidence from the FIDELITY analysis indicates that the efficacy of finerenone is preserved irrespective of concomitant SGLT2 inhibitor or GLP-1 receptor agonist use, with signals of potential additive or synergistic effects on albuminuria reduction, blood pressure lowering, and slowing of kidney function decline [77]. These findings support a “four-pillar” approach to cardiorenal protection, although optimal sequencing, patient selection, and long-term safety of triple or quadruple therapy remain areas of ongoing investigation.

Moreover, RAAS inhibitors represent a foundation of therapy in DKD by reducing intraglomerular pressure and neurohormonal activation; however, residual cardiorenal risk frequently persists, partly driven by ongoing MR activation and the phenomenon of “aldosterone escape.” In this context, finerenone provides complementary blockade of MR-mediated inflammatory and fibrotic pathways, thereby addressing a key pathophysiological mechanism not fully suppressed by RAAS inhibition alone and supporting its use as an add-on therapy [78]. This complementary mechanism translates into additional renoprotective effects and further risk reduction when finerenone is introduced on top of standard therapy [79].

## Conclusions, Comparison with Existing Reviews, and Future Directions: Expanding Indications and Optimizing Combination Strategies

Finerenone represents a major advance within contemporary cardiorenal therapy, supported by robust evidence from the FIDELIO-DKD, FIGARO-DKD, and, more recently, FINEARTS-HF trials, which collectively establish clinically meaningful benefits across the cardiorenal continuum. In patients with T2DM and CKD, these trials consistently demonstrate reductions in both cardiovascular and renal composite outcomes, confirming finerenone as a disease-modifying therapy targeting inflammation and fibrosis through mineralocorticoid receptor antagonism [80]. While earlier reviews have extensively synthesized these data within the specific context of DKD – primarily focusing on phase III trial evidence and its clinical implications [80] – more recent findings suggest that the therapeutic scope of finerenone may extend beyond traditional DKD populations. In particular, emerging evidence from FINEARTS-HF indicates potential benefits in patients with HFmrEF and HFpEF irrespective of diabetes status, although this area remains supported by a comparatively early and still evolving evidence base.

Previous literature has also explored specific dimensions of finerenone efficacy, including subgroup heterogeneity. For instance, analyses stratified by race and kidney function highlight potential variability in treatment response, while also underscoring the limitations of currently available subgroup data and the need for cautious interpretation [81]. In parallel, comparative meta-analyses and network meta-analyses have positioned finerenone alongside other cardiorenal therapies – such as SGLT2 inhibitors and incretin-based agents – demonstrating consistent reductions in kidney composite outcomes and heart failure hospitalizations, while also suggesting differential magnitudes of benefit across drug classes and clinical settings [72, 82].

In this context, combination therapy with SGLT2 inhibitors emerges as a particularly compelling paradigm. Subgroup and pooled analyses consistently show preserved efficacy and acceptable safety with dual therapy, supporting the concept of complementary and potentially synergistic mechanisms: finerenone targeting MR-mediated inflammation and fibrosis, and SGLT2 inhibitors exerting hemodynamic, metabolic, and tubular effects. This integrated approach directly addresses the persistent residual cardiorenal risk observed even under optimized standard of care, a gap repeatedly highlighted across existing reviews [80].

Future research priorities should therefore move beyond single-agent efficacy and focus on optimizing multidrug therapeutic strategies across a broader spectrum of cardiorenal disease. Dedicated randomized trials in non-diabetic CKD are particularly warranted, given the central role of mineralocorticoid receptor overactivation in fibrotic progression independent of glycemic status. In parallel, further investigation is needed to better define the role of finerenone in heart failure populations, where current evidence – although promising – remains less mature compared with DKD. The development of next-generation non-steroidal MR antagonists with enhanced selectivity and improved safety profiles may further refine therapeutic precision.

From a clinical implementation perspective, the translation of trial evidence into real-world practice will require careful patient selection, structured potassium monitoring protocols, and thoughtful integration within existing treatment algorithms that include RAS inhibitors, SGLT2 inhibitors, and, where appropriate, GLP-1 receptor agonists. Ultimately, positioning finerenone within a comprehensive, mechanism-based, and patient-centered therapeutic framework represents a key step toward optimizing long-term cardiorenal outcomes.

Despite the growing body of evidence, several knowledge gaps remain. The lack of direct comparative studies, the reliance on selected populations and subgroup analyses, and the limited availability of long-term real-world data remain key limitations of the evidence, which highlight the need for further investigation. Addressing these limitations will be essential to refine patient selection and optimize the integration of finerenone within evolving multidrug therapeutic strategies.

## Data Availability

Not applicable.
